# Multi-scale habitat modeling framework for predicting the potential distribution of sheep gastrointestinal nematodes across Iran’s three distinct climatic zones: a MaxEnt machine-learning algorithm

**DOI:** 10.1038/s41598-024-53166-1

**Published:** 2024-02-03

**Authors:** Behnam Meshgi, Ahmad Ali Hanafi-Bojd, Saeid Fathi, Galia Modabbernia, Kourosh Meshgi, Mohammad Shadman

**Affiliations:** 1 Department of Parasitology, Faculty of Veterinary Medicine, University of Tehran, P.O.Box 14155-6453, Tehran, Iran; 2 Department of Vector Biology and Control of Diseases, School of Public Health, Tehran University of Medical Sciences, Tehran, Iran; 3 Zoonoses Research Center, Tehran University of Medical Sciences, Tehran, Iran; 4 Department of Parasite Vaccine Research and Production, Razi Vaccine and Serum Research Institute, Agricultural Research, Education and Extension Organization (AREEO), Karaj, Iran; 5 Graduated Biomedical Engineering, Amirkabir University of Technology, Tehran, Iran

**Keywords:** Parasite development, Microbial ecology

## Abstract

Ecological niche models (ENMs) serve as valuable tools in assessing the potential species distribution, identifying crucial habitat components for species associations, and facilitating conservation efforts. The current study aimed to investigate the gastrointestinal nematodes (GINs) infection in sheep, predict and analyze their ecological niches and ranges, and identify the key bioclimatic variables influencing their distribution across three distinct climatic regions in Iran. In a cross-sectional study, a total of 2140 fecal samples were collected from semi-arid (n = 800), arid (n = 500), and humid-subtropical (n = 840) climates in East Azerbaijan, Kerman, and Guilan provinces, respectively. The flotation method was employed to assess stool samples, whereby the fecal egg count (the number of parasite eggs per gram [EPG]) was ascertained for each individual specimen. Employing a presence-only approach, the multi-scale maximum entropy (MaxEnt) method was used to model GINs' habitat suitability using 93 selected points/locations. The findings revealed that Guilan (34.2%) and East Azerbaijan (19.62%) exhibited the utmost proportion of Strongyle-type eggs. East Azerbaijan province also displayed the highest proportion of *Marshallagia* and *Nematodirus*, respectively (approximately 40% and 27%), followed by Guilan and Kerman provinces, while Kerman province had the highest proportion of *Trichuris* (approximately 15%). Ecological niche modeling revealed that the precipitation of the driest quarter (Bio17) exerted the most significant influence on *Marshallagia*, *Nematodirus*, *Trichuris*, and ُSُُُtrongyle-type eggs' presence in East Azerbaijan and Kerman provinces. For Guilan province, the most influential factor defining habitat suitability for Strongyle-type eggs, *Marshallagia*, and *Nematodiru*s was increasing slope. Additionally, the distribution of *Trichuris* was most affected by the variable Bio2 in Guilan province. The study highlights the response of GINs to climate drivers in highly suitable regions, providing insights into ecologically favorable areas for GINs. In conclusion, this study provides a better understanding of GINs and the environmental factors influencing their transmission dynamics.

## Introduction

Gastrointestinal nematodes (GINs) are commonly found in grazing ruminants worldwide and have a detrimental impact on livestock production. These parasites, particularly the trichostrongylid nematode species, are responsible for causing reproductive problems and economic losses in ruminants^1–8^. The most significant nematode species that greatly impact sheep production in temperate climates are *Ostertagia, Teladorsagia*, *Haemonchus*, *Trichostrongylus*, *Nematodirus,* and *Trichuris*^9–11^. Since these parasites have free-living stages, their development, interaction with the host, and transmission dynamics are greatly influenced by different climatic patterns and a variety of environmental situations^12–14^.

In recent years, global warming has posed complex challenges regarding the distribution and severity of certain infectious diseases^15^. The Intergovernmental Panel on Climate Change (IPCC) predicts that there will be further increases in temperature (ranging from 1 to 4 °C) and changes in precipitation are predicted globally^16^. These changes have raised concerns from the Food and Agriculture Organization (FAO) regarding the rise of parasitic diseases in livestock and its potential negative impact on future food security^17^. In many regions, changes in the population dynamics of parasites and the transmission of their free-living stages (such as GINs) are expected due to alterations in land use, the presence of anthelmintic resistance, and extreme climatic changes^14,18,19^. These changes include shifts in seasonal dynamics, species distribution, and an increased risk of habitat loss. Predicting patterns of parasite risk and gaining a better understanding of GINs epidemiology is essential for managing livestock health, particularly in the context of climate change and the strong reliance of the GIN life cycle on environmental conditions, as well as the inadequacy of current nematode control measures^1,6,20–22^.

Ecological niche models (ENMs) and species distribution models (SDMs) are mathematical tools that utilize occurrence records and environmental data to create a correlative model for predicting the ecological requirements and potential geographic distributions of parasites^23,24^. One such model is the maximum entropy (MaxEnt) model, which accurately predicts a species' probability distribution and assesses the impact of climate change on species ranges and the risk of species invasion^25–27^. MaxEnt is a relatively simple machine-learning algorithm with accurate prediction model and stable operation that can handle categorical and continuous environmental layers and small sample sizes, offering the advantage of straightforward result interpretation^28,29^.

Considering the limited information on the effects of climate change and environmental factors on nematode ranges in Iran, as well as the potential risk of species invasions, we employed MaxEnt and Arc-geographical information system (Arc-GIS) platforms to model the potential geographical distribution of GINs in three different climatic regions of Iran: East Azerbaijan, Kerman, and Guilan provinces, representing semi-arid, arid, and humid-subtropical zones respectively. By understanding the geographical distribution of GINs and identifying the dominant bioclimatic and environmental factors affecting their transmission dynamics, we aimed to offer valuable insights to decision-makers in implementing future national surveillance and control measures. Our objective was to predict suitable habitats for GIN species and identify the critical bioclimatic and environmental/topographic variables impacting their geographic distribution.

## Material and method

### Study area

In the present study, sampling was carried out in Guilan, East Azerbaijan, and Kerman provinces which are located in the northern, northwestern, and southeastern regions of Iran, and have humid-subtropical, semi-arid, and arid climatic zones, respectively.

Guilan Province (Coordinates: 37.2774° N 49.5890° E) is situated along the southern strip of the Caspian Sea and spans an area of 14,600 km^2^ with elevations ranging from 15 m below to 300 m above sea level. This province experiences a temperate Caspian climate, with mean monthly temperatures ranging from 6.3 to 29.8 °C, and has the highest rainfall in the region, with a mean annual precipitation of 1506 mm^30^. The large area of Guilan province is mountainous, and covered with dense vegetation^31^.

East Azerbaijan province (Coordinates: 38.0766° N 46.2800° E) is located in the northwest of Iran, and covers an area of about 45,650 km^2^. The climate of this province is characterized by a Mediterranean continental and cold semi-arid climate. The capital city, Tabriz, receives an average annual rainfall of 310 mm^32^.

Kerman province (Coordinates: 30.2907° N 57.0679° E) with an area of about 180,726 km^2^ is situated in the south-central part of Iran. It experiences a semi-arid to dry climate^33^.

### Species occurrence data

**Figure 1:**
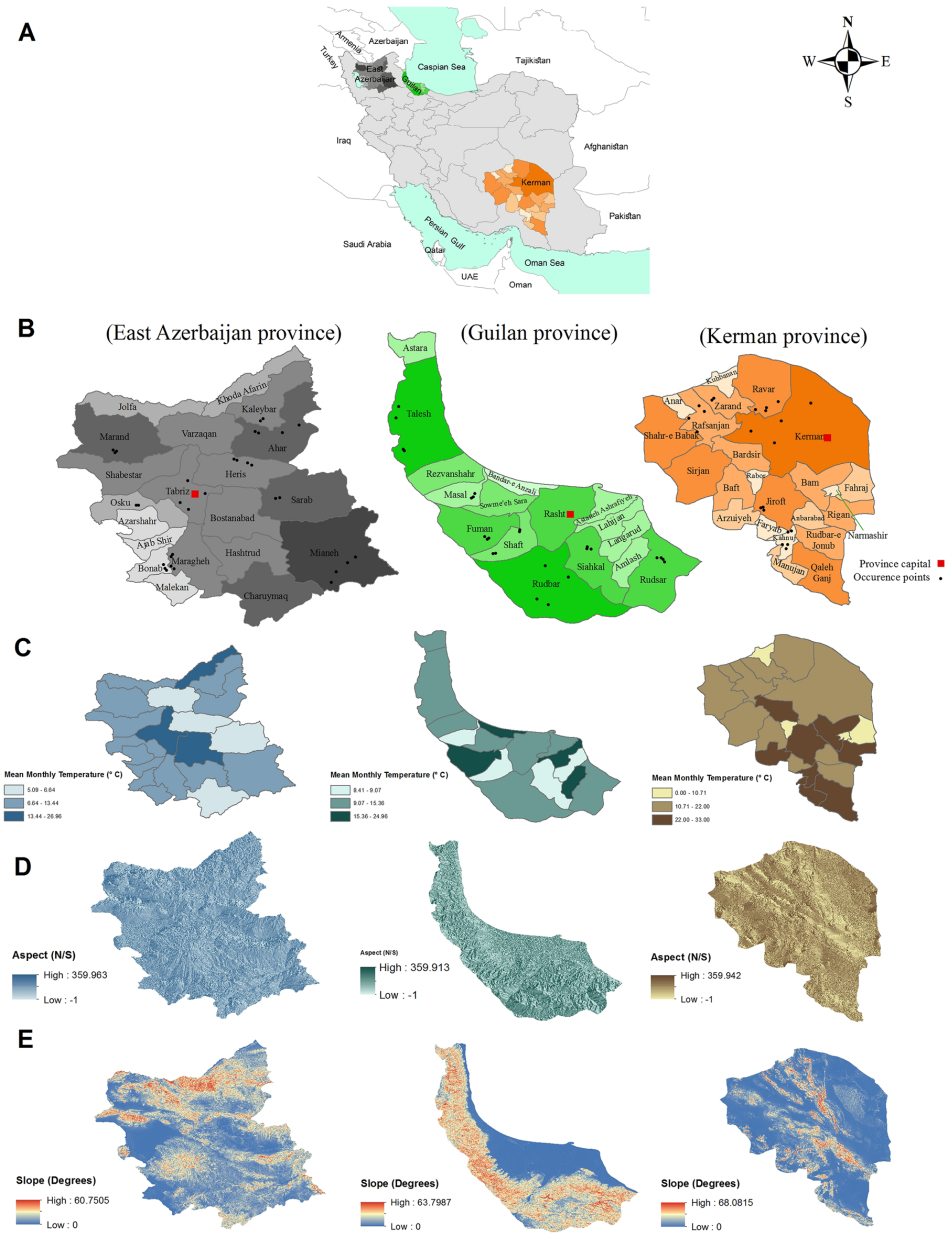
A total of 93 sampling sites were selected as inputs for calibrating the ecological niche models in three provinces (**A**), points/locations are shown on map as black dots (B), as well as three bioclimatic zones. The spatial patterns of some of environmental variables applied in the MaxEnt model were also shown in the figure (**C**–**E**).

A cross-sectional study was conducted from April to September 2020, using cluster sampling. Fresh fecal samples (6 g) were collected directly from the rectum of native sheep using plastic bags which were stored on ice and then refrigerated at 4 °C. A total of 2140 fecal samples were collected from three different climate zones including East Azerbaijan (n = 800), Guilan (n = 840), and Kerman provinces in Iran (n = 500). The study area consisted of ninety-three points/locations, with ten cities and forty villages selected from East Azerbaijan, seven cities and twenty-eight villages selected from Guilan, and six cities and twenty-five villages selected from Kerman (Table 1 and Fig. 1).

**Table 1:** The total volume of samples taken from three climatic zones in the present survey.

Locality		No. of sample	Total number
Climate Zone (Province)	City (Village No.)*		
Semi-Arid (East Azerbaijan)	Tabriz (4)	80	800
	Osku (4)	80	
	Ahar (4)	80	
	Heris (4)	80	
	Bonab (4)	80	
	Maragheh (4)	80	
	Kaleibar (4)	80	
	Miyaneh (4)	80	
	Marand (4)	80	
	Sarab (4)	80	
Humid-Subtropical (Guilan)	Talesh (4)	120	840
	Fuman (4)	120	
	Siahkal (4)	120	
	Rudsar (4)	120	
	Masal (4)	120	
	Shaft (4)	120	
	Rudbar (4)	120	
Arid (Kerman)	Kerman (4)	80	500
	Rafsanjan (4)	80	
	Jiroft (4)	80	
	Zarand (4)	80	
	Kahnuj (5)	100	
	Ravar (4)	80	
Total 23 (93)			2140

* Sampling was performed in 93 villages from 3 different climate zones.

Each labeled sample was sent to the Parasitology Department of the Faculty of Veterinary Medicine at the University of Tehran for identification. The identification process was based on the key morphological characteristics of the nematode's eggs. Additionally, the proportion and intensity of GINs were assessed using the fecal egg count (number of parasite eggs per gram [EPG]). Stool samples were examined microscopically using McMaster's method, with flotation in zinc chloride and saturated salt solution (Specific gravity: 1.53).

### Ecological niche modeling (ENM)

#### Bioclimatic and environmental variables

For the ecological niche modeling, a set of 19 bioclimatic variables, 4 environmental/topographic variables (including aspect [direction of slop], slope, and altitude, as well as the normalized difference vegetation index (NDVI) were used (Figs. 1. and 2, Table 2). The bioclimatic data were extracted from the WorldClim database (http://www.worldclim.org/current) with a spatial resolution of 30 ​s (∼1 ​km^2^).

**Figure 2:**
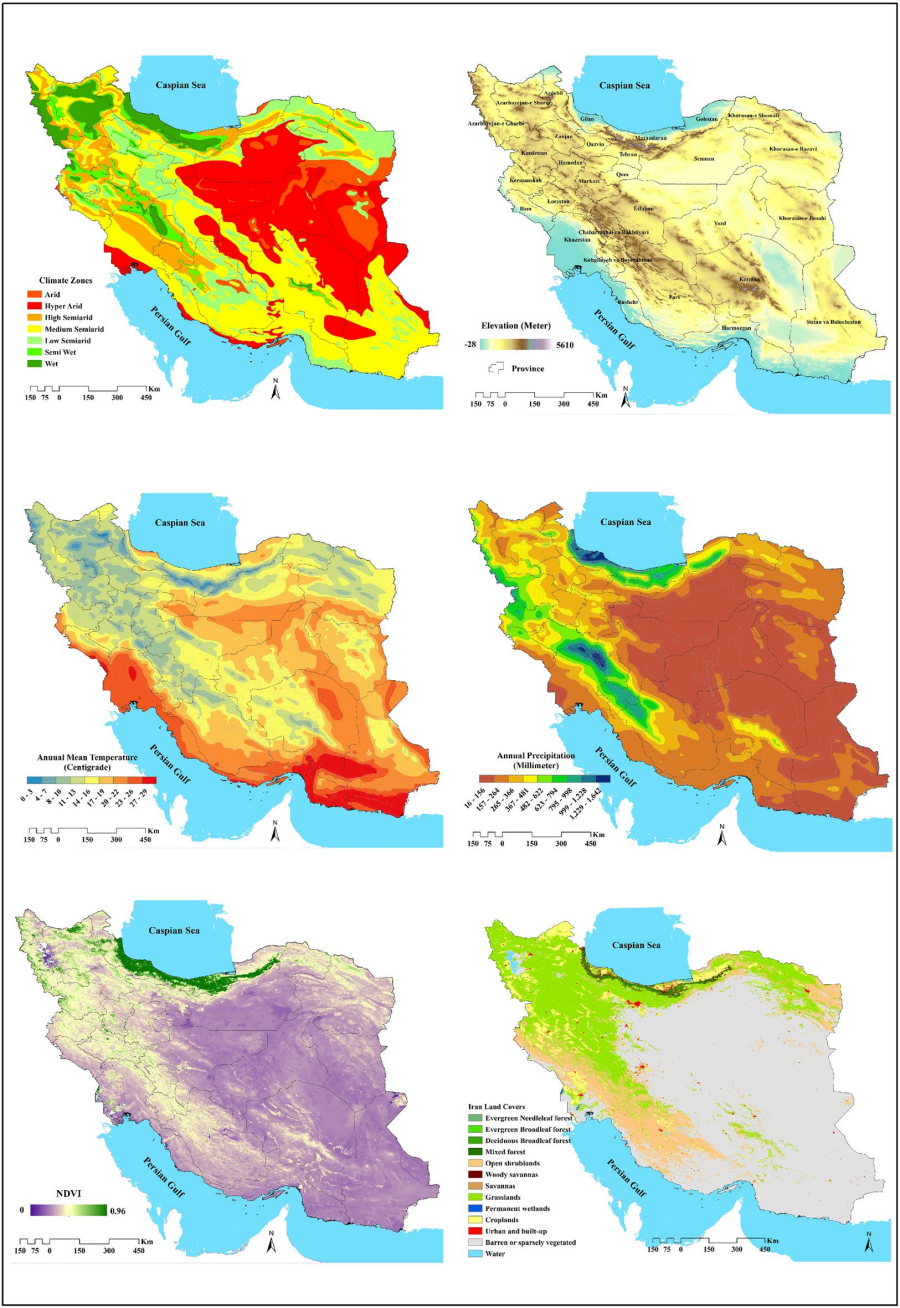
Iran's climatic zones, spatial patterns of the some climatic and environmental variables applied in the MaxEnt model, and Iran's land cover.

**Table 2:** Bioclimatic and environmental variables for predicted model in present study.

Code	Variable (unit)	Code	Variable (unit)
Bioclimatic variables			
Bio 1	Annual mean temperature (^o^C)	Bio 11	Mean temperature of coldest quarter (^o^C)
Bio 2	Mean diurnal range (^o^C)	Bio 12	Annual precipitation (mm)
Bio 3	Isothermality	Bio 13	Precipitation of wettest month (mm)
Bio 4	Temperature seasonality	Bio 14	Precipitation of driest month (mm)
Bio 5	Maximum temperature of warmest month (^o^C)	Bio 15	Precipitation seasonality
Bio 6	Minimum temperature of coldest month (^o^C)	Bio 16	Precipitation of wettest quarter (mm)
Bio 7	Temperature annual range (^o^C)	Bio 17	Precipitation of driest quarter (mm)
Bio 8	Mean temperature of wettest quarter (^o^C)	Bio 18	Precipitation of warmest quarter (mm)
Bio 9	Mean temperature of driest quarter (^o^C)	Bio 19	Precipitation of coldest quarter (mm)
Bio 10	Mean temperature of warmest quarter (^o^C)		
Environmental variables			
	Slope (°)		Altitude (m)
	NDVI		Aspect (°)

Topographic variables layers of aspect and slope were also derived from the digital elevation model (DEM) of Iran obtained from the WorldClim website using ArcMap 10.3. The NDVI data was generated from the MODIS satellite image in 2016 with a spatial resolution of 30 ​s (∼1 ​km^2^) and adapted to an ASCII standard format.

All bioclimatic, topographic and environmental variables were clipped using a boundary mask for Iran boundaries and then adapted to an ASCII standard format in ArcMap 10.5 for later use in MaxEnt modeling.

#### Modeling procedure

The maximum entropy modeling (MaxEnt version 3.3.3 k) was applied to estimate the probability of species presence. MaxEnt was chosen for its good performance in testing the predictive ability of the model^34^. The maxent algorithm is capable of providing the probability distribution of maximum entropy given to empirical constraints such as the spatial distributions of species and environmental conditions, allowing for least-biased, constraint-satisfying probability distribution inference^35^.

Eighty percent of the location points were randomly assigned as model training (model calibration) and the remaining 20% was used to test the model's predictive value with 10 repetitions (model validation), allowing for simple statistical analysis^36^. Maximum iterations were also set to 500.

Habitat suitability was defined by a logistic threshold value (the 10th percentile training presence [TPTP]), ranging from 0 (unsuitable) to 1 (best habitat suitability), to eliminate biologically irrelevant noise in the model^37^.

The accuracy of the modeling (model’s goodness-of-fit) was evaluated using a threshold independent receiver-operating characteristic (ROC) which calculated the area under receiver-operating characteristic curve (AUC) values (0.5 = random to 1.0 = highest value) via plotting the model’s sensitivity against 1- specificity^38^. Following the execution of 10 replicates, the average AUC values were calculated for both the training and test datasets. The contribution of each variable in the model was identified using the percentage contribution of the Jackknife analysis. The ASCII output map of GINs was loaded in ArcGIS 10.5 for the average model.

## Results

### Proportion and intensity of GINs

The findings of the study revealed the most remarkable proportion of Strongyle-type egg (34.2%) and *Trichuris* (14.4%) in Guilan and Kerman provinces, respectively (Table 3)*.* East Azerbaijan province records the highest proportions of *Marshallagia* (39.8%) and *Nematodirus* (27.5%), (Table 3).

**Table 3:** Proportion and intensity of GINs among sheep in three studied climatic zones.

Climate Zone (Province)	City	Point/Location (Sample No.)	Proportion (%) and FEC average							
			Strongyles type		*Marshallagia*		*Nematodirus*		*Trichuris*	
			%	FEC	%	FEC	%	FEC	%	FEC
Semi-Arid (East Azerbaijan)	Tabriz	4 (80)	2.50	1.80	97.50	11.60	61.20	4.70	1.25	1.30
	Sarab	4 (80)	0	0	57.50	5.70	28.70	1.80	0	0
	Marand	4 (80)	6.20	1.30	60	5	51.20	2.40	0	0
	Maragheh	4 (80)	41.20	3.20	45	3.10	41.20	1.80	0	0
	Miyaneh	4 (80)	56.20	7.30	50	2.10	28.70	1.60	1.25	1.40
	Heris	4 (80)	7.50	1.40	25	8	11.20	3.20	1.25	1.20
	Kaleibar	4 (80)	11.20	1	11/20	8.20	18.70	4.90	6.20	1.50
	Ahar	4 (80)	17	1.30	0	0	0	0	0	0
	Osku	4 (80)	27.50	3.50	1	4.20	12.50	7.40	12.50	1
	Bonab	4 (80)	15	5.40	3.50	10	21.20	4	3.70	1.60
	Total mean	40 (800)	19.62	2.12	39.80	6	27.50	3.20	2.60	1.30
Humid-Subtropical (Guilan)	Talesh	4 (120)	27.50	5.50	10	1.60	6.60	1.60	6.60	6.60
	Masal	4 (120)	17.50	2.20	1	1	4.10	1	9.10	5.60
	Siahkal	4 (120)	16.60	2.40	1	1	1.60	1.60	7.50	5.50
	Rudbar	4 (120)	49.10	9.80	5.80	1.10	12.50	1.30	15	8.50
	Rudsar	4 (120)	49.10	15.10	4.10	1	10.80	1.30	8.30	7.90
	Fuman	4 (120)	47.50	7.50	6.60	1.70	3.30	1.20	6.60	3.50
	Shaft	4 (120)	28.30	6.30	1.60	2.10	12.50	1.10	5.80	12.20
	Total mean	28 (840)	34.20	6.70	4.50	1.90	7.30	1.50	8.40	8.10
Arid (Kerman)	Kerman	4 (80)	5	1.50	2.50	1	0	0	20	1.40
	Ravar	4 (80)	2.50	1	2.50	1.40	1.25	1.20	15	1.20
	Zarand	4 (80)	11.20	1.40	1.25	1	0	0	27.50	1.30
	Rafsanjan	4 (80)	13.70	1.60	1.25	1	2.50	1	6.20	1
	Jiroft	4 (80)	12.50	1	13.70	1.60	22.50	1.20	10	1.10
	Kahnuj	5 (100)	35	1.70	3	1.30	14	1	9	1
	Total mean	25 (500)	14.20	1.70	4.40	1.20	7	1	14.40	1.40

In Guilan province, specifically in 7 cities and 28 villages, the proportion of Strongyle-type egg was by far the highest (34.2%), followed by *Trichuris* (8.4%), *Nematodirus* (7.3%), and *Marshallagia* (4.5%).

East Azerbaijan province exhibited the highest proportion of *Marshallagia* (39.8%), followed by *Nematodirus* (27.5%), Strongyle-type egg (19.62%), and *Trichuris* (1.3%).

Kerman province was found to harbor *Trichuris* (14.4%) with the highest proportion, followed by Strongyle-type egg (14.2%), *Nematodirus* (7%), and *Marshallagia* (4.4%).

The overall intensity of GINs across the different climates was found to be low, ranging from 1 to 8.1 based on EPG (range; 1–8.1), (Table 3).

### Species distribution modeling and contribution of variables

**Figure 3:**
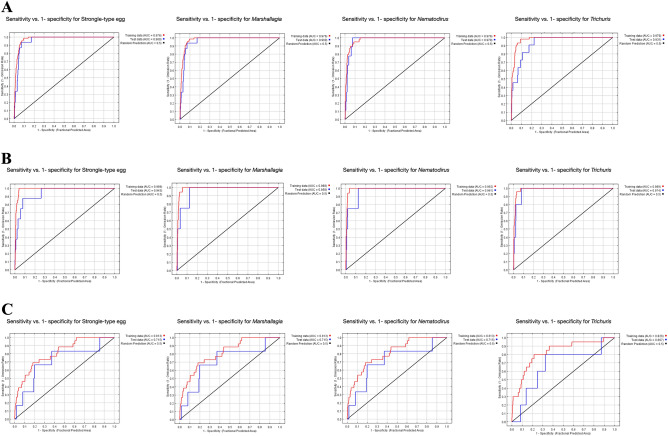
Model response curves for the area under the curve for gastrointestinal nematodes of sheep in three climates zone, Iran. Training test and random prediction AUC values provided by performed models in the modeling analysis. (**A**) Azerbaijan province, (**B**) Kerman province, (**C**): Guilan province.

**Figure 4:**
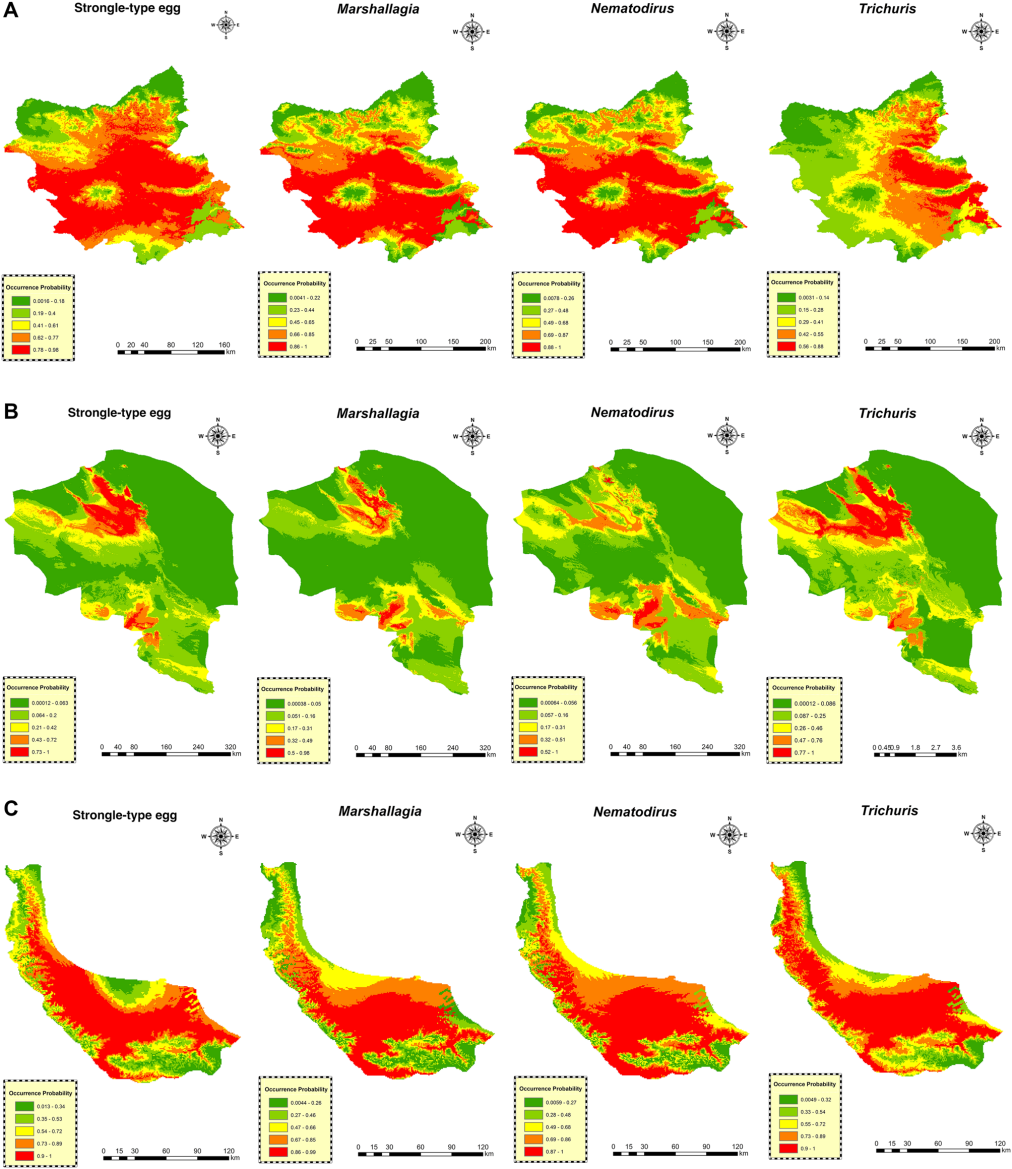
MaxEnt habitat suitability maps of GINs in three climatic regions, Iran. Areas depicted as red are of high suitability and areas depicted as blue are of low suitability. Areas depicted as green to red show suitability of regions from 0 (unsuitable habitat) to 1 (highly suitable habitat), visualizing the potential risk of GINs. (**A**): Azerbaijan province, (**B**): Kerman province, (**C**): Guilan province.

Models constructed with variables demonstrated relatively accurate prediction. The plotted ROC curve displayed the AUC values ranging from 0.81 to 0.99 for the training data and from 0.66 to 0.97 for the test data (Fig. 2). The AUC train and AUC test for each species are denoted separately in Fig. 3, while Fig. 4 depicts the probability maps showing species occurrence predictions arranged by the Maxent color scheme, ranging from green (occurrence “0”) to red (occurrence “1”).

**Figure 5:**
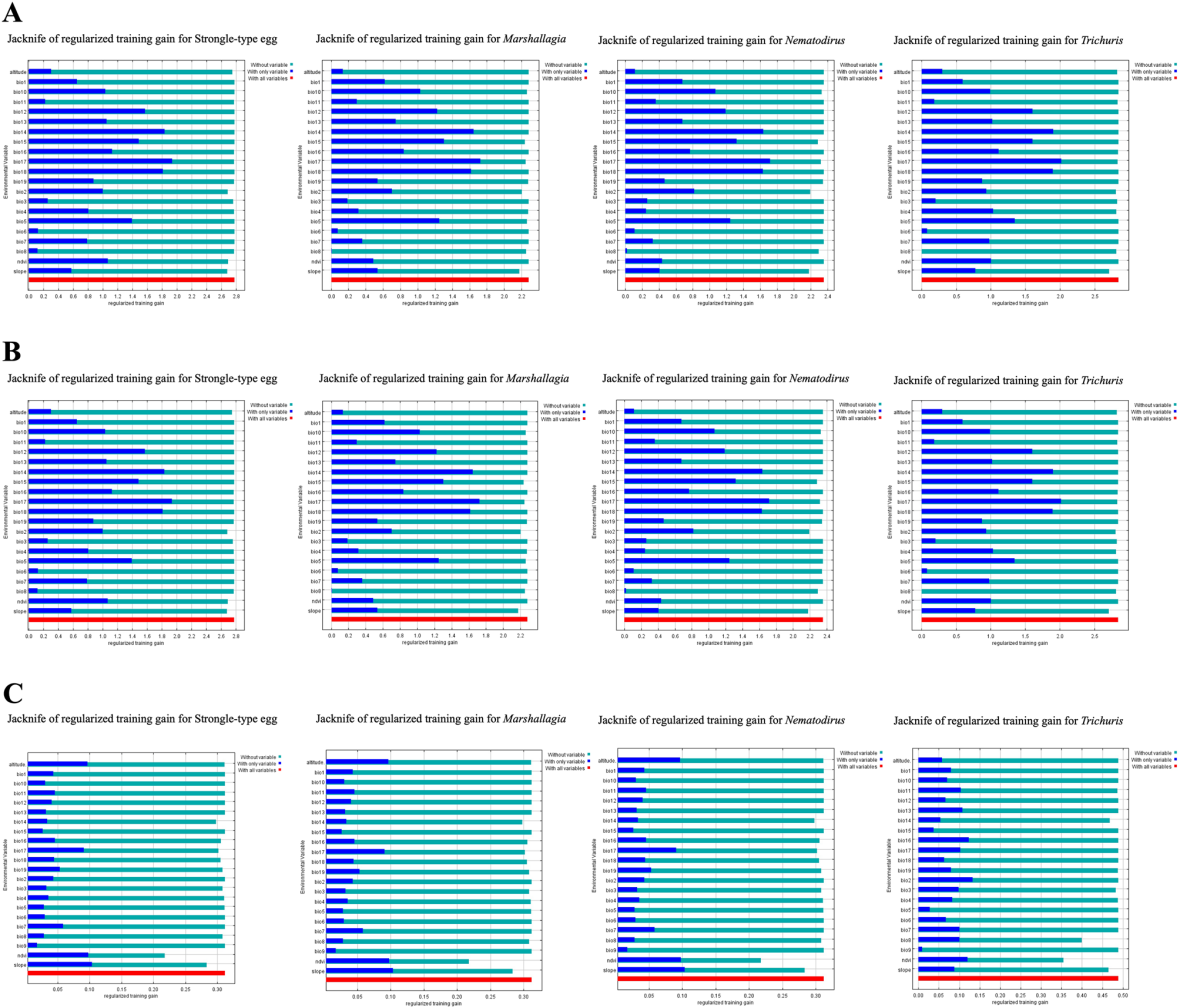
The Jackknife test for environmental variables’ contribution in modeling in three climates zone, Iran. Training and AUC gains is based upon the three scenarios including without variable, with only variable and with all variable. Blue bar depicts regularized training gain of individual environmental variable relative to all environmental variables (Red bar). (**A**) Azerbaijan province, (**B**) Kerman province, (**C**) Guilan province.

Through the MaxEnt model's internal jackknife analysis, it was revealed that three variables of Bio17, Bio14, and Bio18 have the highest mean contributions in predicting the distribution of *Marshallagia*, *Nematodirus*, *Trichuris*, and Strongyle-type eggs in East Azerbaijan and Kerman provinces when used in isolation (Fig. 5). In Guilan province, slope, NDVI, and altitude were the variables with the highest importance contribution when used in isolation for *Marshallagia, Nematodirus,* and Strongyle-type eggs. Moreover, the jackknife test of variables' contribution indicated that Bio2, Bio16, and NDVI were the most significant predictors of the presence probability of *Trichuris* in Guilan province (Fig. 5).

### Response of variables to GINs habitat suitability

**Figure 6:**
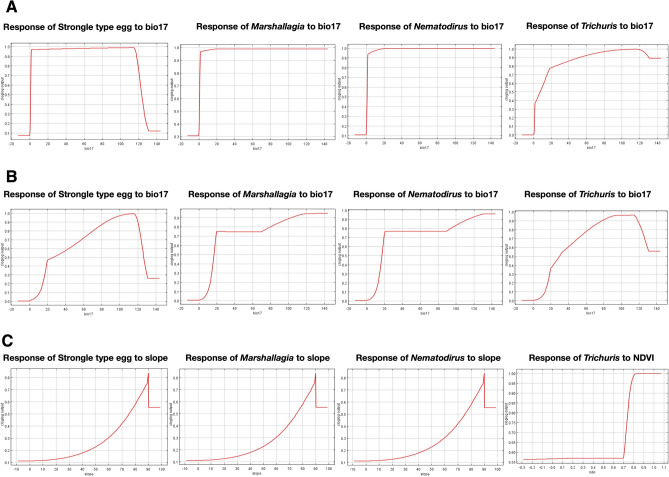
Response curves of MaxEnt models for dominant variables; Bio17 (Precipitation of the driest quarter; mm), slope and NDVI contributed remarkably to the suitability model, indicating the most significant influence of variables on presence-only habitat suitability of GINs. (**A**): East Azerbaijan province, (**B**): Kerman province, (**C**): Guilan province.

Figure 6 presents the response curves of the most important variables to GINs habitat suitability.

Our model demonstrated that precipitation of the driest quarter (Bio17) plays a crucial role in controlling the distribution of *Marshallagia*, *Nematodirus*, *Trichuris,* and Strongyle-type eggs in East Azerbaijan and Kerman provinces. Additionally, slope was identified as a key topographic variable influencing the distribution of Strongyle-type eggs, *Marshallagia*, and *Nematodirus* in Guilan province, while Bio2 had the most significant impact on the presence probability of *Trichuris* in this province. It is noteworthy that NDVI exhibited the largest decrease in value when removed, (lighter blue bars), suggesting that it incorporates valuable information not available in the other variables.

## Discussion

Among the three climatic regions under study, the humid-subtropical area (Guilan province) exhibited the highest proportion of Strongyle-type eggs (34.2%). This was followed by the Mediterranean continental and the cold semi-arid climate (East Azerbaijan province) with a proportion of 19.62% and arid area (Kerman province) with a proportion of 14.2%. The EPG, on the other hand, was found to be lower in all climatic zones under investigation, ranging from 1 to 8.1.

Whereas, the East Azerbaijan province showed the highest proportion of *Marshallagia* (39.8%) and *Nematodirus* (27.5%), surpassing the other two regions considerably. The remaining two zones, Guilan and Kerman, made manifest a proportion tantamount to less than 8% and 5% of the aforementioned parasites, respectively. Furthermore, the highest proportion of *Trichuris* eggs in sheep (14.4%) was observed in Kerman province compared to the wet province of Guilan and East Azerbaijan province.

To compare the infection rates of sheep GINs between older reports dating back more than four decades and the present day, it is evident that not only are the rates of GINs on the decline but also EPG is on the decline presently^39,40^. This decline in GINs is occurring, owing to the increasing implementation of anthelmintic treatments and sheep movement, which suggests a positive trend in herd management strategies.

Ruminant GINs are greatly influenced by environmental and climatic conditions. The main parasitic pathogens of the ruminant’s gastrointestinal tract such as *Haemonchus*, *Ostertagia*, *Teladorsagia*, *Trichostrongylus*, *Nematodirus*, *Oesophagostomum*, and *Trichuris* play a crucial role in animal health^11,41,42^.

Climatic variables have the potential to influence the prevalence, intensity, and geographical distribution of GINs, particularly when parasite species exhibit climate-driven spatial variation. Temperature and rainfall, being the foremost influential factors, exert a significant impact on the growth and development of free-living stages, including eggs and various larval stages (namely, L1, L2, L3) of these nematodes ^6,11^.

Additionally, climate change holds the capacity to exert its influence upon the transmission success of parasites by altering the host immune defenses, alongside altering the density of these hosts, thus irrevocably impacting the spatial distribution of these species^43,44^. By establishing a correlation model that demonstrates the connection between species and environmental and bioclimatic variables, important insights into species' ecological requirements and habitat suitability can be obtained^24^.

In the present study, the modeling results signify that GINs are distributed to diverse extents across nearly all territories of East Azerbaijan, except for *Trichuris*, which is predominantly found in the western regions of the province. The jackknife analysis revealed that Bio17 had the most significant contribution to the model, followed by Bio14 and Bio18, in determining the habitat suitability of GINs in East Azerbaijan. This province experiences a Mediterranean continental and cold semi-arid climate.

In Kerman province, the modeling results revealed that the main distribution of GINs is concentrated in the southern and northern regions of the province. The jackknife analysis predicted Bio17 as the variable with the most contribution in the model for all nematodes under investigation in the province, followed by Bio 14 and Bio18. The southeastern fringes of Iran, including Kerman province, are particularly affected by the monsoon weather phenomenon. With a semi-arid to dry climate characterized by low rainfall and high temperatures, the more arid regions create unfavorable conditions for larval development, survival, and migration out of feces onto herbage. Consequently, these regions provide less environmentally suitable habitats for GINs.

In the province of Guilan, the presence of distribution of GINs spans across nearly all areas, indicating that the conditions are highly favorable for the growth and development of eggs and free-living stages of trichostrongylid species. The distribution of Strongyle-type eggs, *Marshallgia*, *Nematodirus*, and *Trichuris*, does also bear close resemblance. The predicted factors with the most significant influence on *Marshallagia*, *Nematodirus*, and Strongyle-type eggs are envisaged to be the slope, followed by NDVI, and altitude. Additionally, we have discovered that three variables, namely Bio2, Bio16, and NDVI, are the most important predictors of the presence probability of *Trichuris*.

In the northern regions of Iran, specifically the Caspian and Wet Mediterranean zones, a humid-subtropical climate prevails, with mild rainy winters and humid hot summers. These areas tend to experience fewer dry periods, thus creating suitable environmental conditions for the development of eggs and free-living stages of GIN species.

During winter, third-stage larvae (L3) of most species are capable of residing in the rangelands, resulting in the infection of ruminants, particularly newborn lambs, during the spring season^45^. The monthly rainfall in Rasht and Bandar-e Anzali within Guilan province, excluding the summer season, bestows abundant moisture essential for both the robust development of trichostrongylid larvae in fecal pellets and the successful outward migration of L3 larvae from feces. However, it is not conducive to the vertical migration of L3 onto herbage^31,45,46^. This condition may also be present, to a lesser extent, in East Azerbaijan province during the rainy season (autumn, winter, and spring). On the other hand, this condition is not widespread in climate regions such as Kerman province, characterized by ''Hot Dry Desert and Hot Semi-Desert'' climates, thereby limiting the possibilities of migration out of feces and subsequent survival.

In the analysis of species' spatial distribution and their climate-driven spatial variations, it is of utmost importance to refrain from disregarding the finer-scale geographic variations, including the impact of altitude. The Caspian region proved altitude's substantial role as a determining factor for the model of *Marshallagia* and *Nematodirus*.

Eggs and free-living stages of trichostrongylid infections such as *Trichostrongylus*, *Ostertagia*, *Nematodirus*, and *Cooperia* showcase an ability to survive at very low temperatures (e.g., above 4 °C for the development of L3 larvae). However, *Haemonchus* requires temperatures above 8 °C for L3 larvae development. As temperatures rise in spring, pasture contamination with L3 larvae of most species increases throughout the summer, extending into autumn for certain species. Milder winters can facilitate the over-winter survival of *Haemonchus* L3^21^. Conversely, extremely high temperatures may pose unsuitability for larval survival, hindering their feeding and increasing metabolism^6,13,21,47^. Warm temperatures have been associated with a significant decrease in *Ostertagia* spp. The eggs and free-living larvae of *Ostertagia* and *Nematodirus* exhibit resistance to cold temperatures, and snow cover plays a protective role for infective larvae, however, severe winters are connected to a decrease in endemic species^47^. Drier summers could reduce grass growth and availability of summer grazing, inhibiting larvae development^6^. It is worth noting that the shedding of eggs by adult worms during the periparturient egg rise period results in the widespread distribution of large quantities of eggs in the pasture after immunity to trichostrongylid infection. *Nematodirus* spp. infection is not related to the periparturient egg rise in ewes; instead, lambs are responsible for the contamination of pastures. Furthermore, *Nematodirus* eggs have been found to require warmth in spring after prolonged chilling (below 11 °C) for their development^47,48^.

From a local perspective, bioclimatic variables such as temperature, rainfall, and their variations within and between years, along with topographic/vegetation variables as well as their interactions with husbandry and management practices (farm-level risk factors), are all implicated in larval availability and the geographical distribution of the parasites, however, a strategic dosing regimen, consisting of frequent administration of anthelmintic drugs, has been widely employed in the country over the past decades to reduce infection rates. This approach, devoid of comprehensive understanding of GINs population dynamics, may precipitate the manifestation of anthelmintic resistance. Instead, a more regionally attuned strategy, acknowledged as targeted treatment, ought to be adopted. At a regional level, ecological niche models hold great value in predicting primary infection periods, assessing the impact of intervention impacts, and understanding the influence of environmental and bioclimatic factors. These models aid in the formulation of rational strategies such as judicious use of anthelmintics, to effectively reduce parasite transmission and alleviate the burden on animals, while mitigating economic damage.

## Conclusions and further recommendations

In the present study, MaxEnt employed an ecological niche modeling technique to generate a habitat suitability map for GINs in the provinces of East Azerbaijan, Guilan and Kerman, Iran. The objective was to elucidate the primary predictors of GINs presence probability across various host environments, incorporating bioclimatic and environmental variables.

Regarding all nematodes under examination (*Marshallagia*, *Nematodirus*, *Trichuris*, and Strongyle-type eggs), the variable Bio17 exhibited the most substantial contribution to the model in East Azerbaijan and Kerman provinces, followed closely by Bio14 and Bio18. In Guilan province, the variables with the greatest impact on *Marshallagia*, *Nematodirus*, and Strongyle-type eggs' presence probability were slope, subsequently, followed by NDVI, and altitude. In the humid-subtropical climate of Guilan province, the paramount determinants showcasing the presence probability of *Trichuris* are evidently Bio2, Bio16, and NDVI.

Further, it is of paramount significance to engage in comprehensive research pertaining to the ecological niche of ruminant GINs not solely within the particular areas being investigated, but also spanning diverse regions nationwide. Such investigations should specifically prioritize the analysis of seasonal dynamics and herd-level management factors in relation to the predictive model.

## Data Availability

All data generated are included in the current article.

## Abbreviations

GINs: Gastrointestinal nematodes
SDMs: Species distribution models
MaxEnt: Maximum entropy
Arc-GIS: Arc-geographical information system
EPG: Number of parasite eggs per gram
DEM: Digital elevation model
ROC: Receiver-operating characteristic
AUC: Area under receiver-operating characteristic curve
FAO: Food and agriculture organization
FEC: Fecal egg count
ENMs: Ecological niche models
NDVI: Normalized difference vegetation index
